# Supplementary material for: Seriniquinones as Therapeutic Leads for Treatment of BRAF and NRAS Mutant Melanomas
Source: Molecules. 2021 Dec 4;26(23):7362. doi: 10.3390/molecules26237362 (PMC8658889; doi:10.3390/molecules26237362)
Supplement: Supplementary file 1 [file molecules-26-07362-s001.zip › molecules-1459668-supplementary.pdf]

Table S1: IC_{50} values for seriniquinone (SQ1) and its synthetic analogs for 72h treatment determined by MTT assay on cell lines (n=3).

Compounds		SK-MEL-28	SK-MEL-19	SK-MEL-147	WM293A	501mel	MM200	MCF7	HCT-116	MRC-5	FDH
SQ1	**IC_{50} (µM)**	0.15	0.06	0.13	1.05	0.32	1.36	3.3	1.0	0.64	1.15
	**95% CI**	n.d.	0.05 – 0.09	0.08 – 0.21	0.73 – 1.49	0.27 – 0.39	0.1 – 1.85	2.9 – 3.7	n.d.	0.32 – 1.27	n.d.
	**R^2**	0.96	0.89	0.86	0.79	0.94	0.81	0.92	0.89	0.87	0.95
SQ2	**IC_{50} (µM)**	0.04	0.04	0.1	0.74	0.11	0.75	0.73	0.23	1.57	1.17
	**95% CI**	n.d.	0.03 – 0.06	0.07 – 0.15	0.51 – 1.08	0.05 – 0.24	0.6 – 0.93	0.63 – 0.84	n.d.	0.83- 2.95	n.d.
	**R^2**	0.92	0.9	0.87	0.8	0.9	0.92	0.97	0.92	0.85	0.92
SQ3	**IC_{50} (µM)**	0.24	0.11	0.26	1.5	0.2	1.43	1.9	1.02	1.43	3.5
	**95% CI**	n.d.	0.06 – 0.2	0.15 – 0.44	1.12 – 1.97	n.d.	1.19 – 1.72	1.6 – 2.3	n.d.	1.01 – 2.63	n.d.
	**R^2**	0.9	0.8	0.75	0.84	0.62	0.95	0.92	0.88	0.96	0.94
SQ4	**IC_{50} (µM)**	0.19	0.32	0.4	1.75	>5	1.16	1.0	1.02	2.45	1.01
	**95% CI**	0.14 – 0.27	0.24 – 0.42	0.26 – 0.61	1.32 – 2.31	n.d.	n.d.	0.89 – 1.17	n.d.	1.36 – 4.41	n.d.
	**R^2**	0.83	0.9	0.8	0.85	0.7	0.9	0.95	0.87	0.86	0.94
SQ5	**IC_{50} (µM)**	0.15	0.04	0.07	1.65	1.71	0.3	0.49	0.22	0.56	0.93
	**95% CI**	0.09 – 0.24	0.03 – 0.06	0.05 – 0.11	1.3 – 2.1	n.d.	n.d.	0.41 – 0.57	n.d.	0.37 – 0.85	n.d.
	**R^2**	0.86	0.84	0.74	0.9	0.73	0.97	0.95	0.97	0.96	0.94
DOX	**IC_{50} (µM)**	1.22	0.39	0.18	0.06	0.26	0.1	0.56	0.15	0.05	0.78
	**95% CI**	0.83 – 1.8	0.23 – 0.66	0.12 – 0.29	0.04 – 0.08	0.2 – 0.35	0.07 – 0.13	0.11 – 0.3	0.1 – 0.22	n.d.	0.85 – 2.06
	**R^2**	0.94	0.85	0.94	0.92	0.93	0.96	0.95	0.96	0.48	0.93

LEGEND: n.d. = not determinated; CI = confidence intervals (µM); DOX = doxorubicin.

Table S2: IC_{50} values for seriniquinone (SQ1) and its synthetic analog 2 (SQ2) by MTT assay on melanoma cell lines SK-MEL-28 and SK-MEL-147 in different times of treatments (n=3).

		SK-MEL-28			*SK-MEL-147*		
		**24h**	**48h**	**72h**	**24h**	**48h**	**72h**
SQ1	**IC_{50} (µM)**	**0.56**	**0.69**	**0.15**	**1.79**	**1.61**	**0.13**
	**95% CI**	0.42 – 0.74	0.55 – 0.86	n.d.	1.36 – 2.34	1.19 – 2.16	0.08 – 0.21
	**R^2**	0.93	0.94	0.96	0.89	0.80	0.86
SQ2	**IC_{50} (µM)**	**0.14**	**0.26**	**0.04**	**1.26**	**0.89**	**0.1**
	**95% CI**	0.1 – 0.19	0.19 – 0.36	n.d.	0.83 – 1.93	0.76 – 1.03	0.07 – 0.15
	**R^2**	0.94	0.87	0.92	0.83	0.94	0.88
DOX	**IC_{50} (µM)**	**1.39**	**2.11**	**1.22**	**0.79**	**0.48**	**0.18**
	**95% CI**	0.98 – 1.97	1.59 – 2.81	0.83 – 1.78	0.46 – 1.34	0.27 – 0.87	0.12 – 0.29
	**R^2**	0.86	0.91	0.94	0.93	0.87	0.94

LEGEND: n.d. = not determined; CI = confidence intervals (µM); DOX = doxorubicin.

Table S3: Primer sequences and concentrations for quantitative PCR.

Gene	Sequence (5’ → 3’)	Concentration
DCD	FW: AAGCCAAGGAAGCAGAGATCC<br>RV: GCTCCTTTACCCACGCTTTCT	300 nM
BCL2	FW: ATGTGTGTGGAGAGCGTCAA<br>RV: ACAGTTCCACAAAGGCATCC	300 nM
BCL2L1	FW: CTTGGATGGCCACTTACCTGAA<br>RV: GCTGCTGCATTGTTCCCATA	300 nM
MCL1	FW: GTAATAACACCAGTACGGACGG<br>RV: TCCCGAAGGTACCGAGAGAT	300 nM
BAX	FW: GAGCTGCAGAGGATGATTGC<br>RV: CAGCTGCCACTCGGAAAA	300 nM
BAK1	FW: TGAGTACTTCACCAAGATTGCCA<br>RV: AGTCAGGCCATGCTGGTAGAC	300 nM
BAD	FW: CACCAGCAGGAGCAGCCAAC<br>RV: CGACTCCGGATCTCCACAGC	300 nM
BCL2L11	FW: ATGTCTGACTCTGACTCTCG<br>RV: CCTTGTGGCTCTGTCTGTAG	300 nM
BID	FW: ATGGACCGTAGCATCCCTCC<br>RV: GTAGGTGCGTAGGTTCTGGT	300 nM
PMAIP1	FW: CGCGCAAGAACGCTCAACC<br>RV: CACACTCGACTTCCAGCTCTGCT	300 nM
BBC3	FW: GACCTCAACGCACAGTACGAG<br>RV: AGGAGTCCCATGATGAGATTGT	300 nM
BNIP3	FW: ATATGGGATTGGTCAAGTCGG<br>RV: CGCTCGTGTTCCTCATGCT	300 nM
BECN1	FW: TCTGAAGAGGACCTGGACCCT<br>RV: GGCTCACGTCCATCTCGTC	300 nM
ATG5	FW: GGGCCATCAATCGGAAAC<br>RV: AGCCACAGGACGAAACAG	300 nM
ATG7	FW: CGTTGCCCACAGCATCATCTTC<br>RV: TCCCATGCCTCCTTTCTGGTTC	300 nM
MAP1LC3B	FW: AAGGCGCTTACAGCTCAATG<br>RV: CTGGGAGGCATAGACCATGT	300 nM
XIAP	FW: GACAGTATGCAAGATGAGTCAAGTCA<br>RV: GCAAAGCTTCTCCTCTTGCAG	300 nM
BIRC5	FW: GCCCAGTGTTTCTTCTGCTTCA<br>RV: GCACTTTCTCCGCAGTTTCCTC	300 nM
HPRT1	FW: GAACGTCTTGCTCGAGATGTGA<br>RV: TCCAGCAGGTCAGCAAAGAAT	300 nM
ACTB	FW: AGGCCAACCGCGAGAAG<br>RV: ACAGCCTGGATAGCAACGTACA	300 nM
RPLPO	FW: GCAATGTTGCCAGTGTCTG<br>RV: GCCTTGACCTTTTCAGCAA	300 nM

FW: Forward; RV: Reverse.

Table S4: IC_{50} (µM) of seriniquinone (SQ1), its synthetic analog 2 (SQ2), rapamycin and doxorubicin by MTT assay in SK-MEL-28 and SK-MEL-147 melanoma cell lines of 24h treatment. Cells were 1h pre-treated with 30 nM bafilomycin A1 and 20 µM Z-VAD-FMK (n=3).

**SK-MEL-28**						
Combinations	**-**		***BAFILOMYCIN A1***		***Z-VAD-FMK***	
RAPAMYCIN	17.75		15.65		31.79	
R^2 / CI 95%	0.80	11.0-28.5	0.39	8.9-27.4	0.75	25.4-39.8
DOXORUBICIN	0.59		0.33		1.15	
R^2 / CI 95%	0.95	0.5-0.7	0.92	0.27-0.4	0.89	0.89-1.47
SQ1	0.35		0.14		0.7	
R^2 / CI 95%	0.95	0.3-0.4	0.92	0.1-0.2	0.84	0.5-0.9
SQ2	0.24		0.06		0.37	
R^2 / CI 95%	0.96	0.2-0.3	0.95	0.05-0.08	0.89	0.3-0.5
**SK-MEL-147**						
Combinations	**-**		***BAFILOMYCIN A1***		***Z-VAD-FMK***	
RAPAMYCIN	17.47		39.1		22.8	
R^2 / CI 95%	0.89	11.0-27.8	0.69	n.d.	0.87	17.1-30.3
DOXORUBICIN	0.8		2.48		1.88	
R^2 / CI 95%	0.87	0.6-1.1	0.51	1.3-4.7	0.73	1.3-2.8
SQ1	1.64		2.26		1.89	
R^2 / CI 95%	0.78	1.2-2.3	0.44	1.3-4.0	0.72	1.4-2.6
SQ2	0.91		1.18		1.28	
R^2 / CI 95%	0.86	0.7-1.2	0.55	0.5-2.6	0.7	0.9-1.8

LEGEND: CI = confidence intervals (µM).

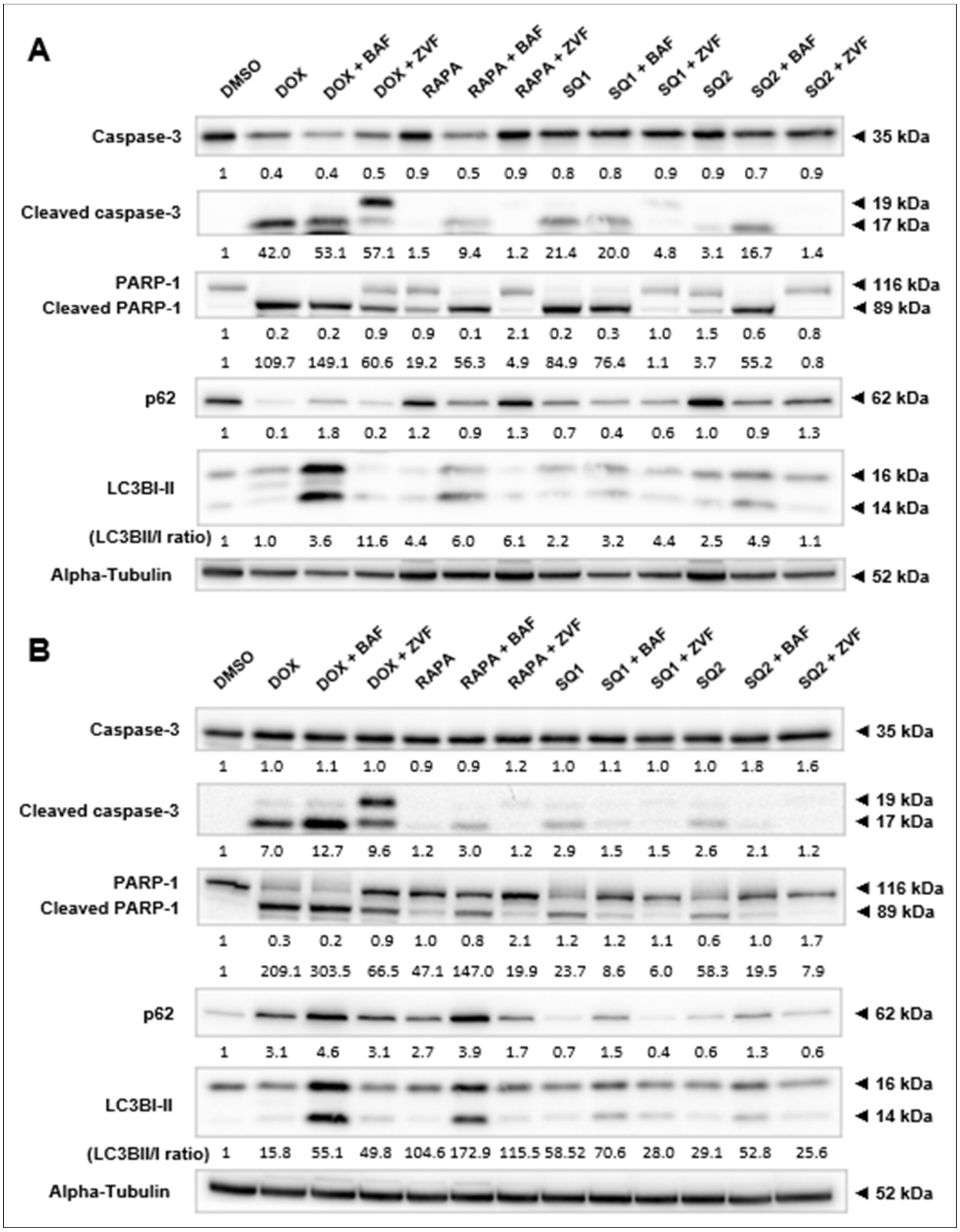

Figure S1: Quantifications from Western blotting analysis with inhibitor treatments. SK-MEL-28 **(A)** and SK-MEL-147 **(B)** were submitted to 1h pre-treatments of 30 nM bafilomycin A1 (BAF) and 20 µM Z-VAD-FMK (ZVF). Protein expression (PARP-1, cleaved PARP-1, caspase 3 and cleaved caspase 3, p62 and LC3B) was evaluated by western blotting after 24h of incubation with: DMSO (negative control: 0.2%), doxorubicin - DOX (positive control: 1.4 µM for SK-MEL-28 and 0.8 µM for SK-MEL-147), rapamycin - RAPA (18 µM), **SQ1** (0.6 µM for SK-MEL-28 and 1.79 µM for SK-MEL-147) and **SQ2** (0.1 µM for SK-MEL-28 and 1.26 µM for SK-MEL-147). Quantifications were determined by the UN-SCAN-IT Gel 6.1 (Silk Scientific), normalized by alpha-tubulin and compared with the negative control of each experiment, showing the mean values (n=3).
